# Influencing factors of three pronuclei incidence and their impact on pregnancy outcomes in women with good prognosis undergoing conventional *in vitro* fertilization with donor sperm: a retrospective cohort study

**DOI:** 10.3389/frph.2025.1509710

**Published:** 2025-03-13

**Authors:** Jianhua Sun, Xiang Liu, Shengjia Shi, Mingzhao Li

**Affiliations:** The ART Center, Northwest Women’s and Children’s Hospital, Xi'an, China

**Keywords:** three pronuclei, conventional *in vitro* fertilization, donor sperm, infertility, pregnancy outcomes, live birth rate

## Abstract

**Purpose:**

This study aimed to investigate the influencing factors of three pronuclei (3PN) zygote incidence and their impact on pregnancy outcomes in women with good prognosis undergoing conventional *in vitro* fertilization with donor sperm (C-IVFD).

**Methods:**

This retrospective study included women aged 35 years or younger who underwent the long/ultra-long follicular phase agonist protocol between January 2014 and January 2021. C-IVFD cycles were divided into the 3PN = 0% group (no 3PN zygotes) and the 3PN > 0% group (with 3PN zygotes). Multivariate logistic regression analysis was performed to identify factors influencing 3PN zygote incidence. The primary outcomes were clinical pregnancy, ongoing pregnancy, abortion and live birth rates. The secondary outcomes were cleavage, high-quality embryo, available embryo, implantation and ectopic pregnancy rates.

**Results:**

1,250 embryo transfer cycles were included in this study. The peak estradiol (E_2_) level on the day of human chorionic gonadotrophin (hCG) administration (OR: 1.16, 95% CI 1.12–1.19, *p* < 0.001) and the number of retrieved oocytes (OR: 1.08, 95% CI 1.05–1.11, *p* < 0.001) were independently associated with 3PN incidence. Compared to the 3PN > 0% group, the 3PN = 0% group exhibited significantly higher ongoing pregnancy rates (*p* = 0.033) and live birth rates (*p* = 0.009), as well as lower abortion rate (*p* = 0.026). No significant differences were found between the 3PN = 0% and 3PN > 0% groups in cleavage, high-quality embryo, available embryo, implantation and ectopic pregnancy rates (*p* > 0.05).

**Conclusions:**

The peak E_2_ level on hCG administration day and the number of retrieved oocytes were independently associated with 3PN incidence. The incidence of 3PN zygotes has a negative impact on pregnancy outcomes in women with good prognosis undergoing C-IVFD.

## 1 Introduction

Infertility impacts roughly 15% of couples globally, with male factors contributing to 20%–30% of cases and female factors accounting for 20%–35%. Furthermore, a combined contribution from both factors is implicated in 25%–40% of infertility instances. It is noteworthy that there has been a steady increase in the demand for fertility treatments utilizing donor sperm in recent years (1–3). The quality of semen is influenced by various factors, including the male's own health status, lifestyle habits, and age. Donor sperm sourced from nationally approved and operated human sperm banks undergo rigorous screening and testing, ensuring their quality and quantity. Studies have demonstrated that the risk of adverse pregnancy outcomes does not increase when using donor sperm compared to using partner sperm (1, 4, 5). The utilization of donor sperm results in a decrease in the rates of miscarriage and ectopic pregnancy (5), while simultaneously increasing the rates of live births (6). In China, the primary reasons for utilizing donor sperm for assisted reproduction are azoospermia and failures in intracytoplasmic sperm injection (ICSI).

In the process of *in vitro* fertilization (IVF)—embryo transfer, normal fertilization is defined as the appearance of two distinct pronuclei (PN) within the oocyte 16–20 h post-fertilization, accompanied by the presence of two polar bodies. Conversely, cases of a single pronucleus (1PN), three pronuclei (3PN), multiple pronuclei (multiple PN), and the absence of pronuclei (0PN) in fertilized oocytes are considered abnormal fertilizations and are discarded. Although studies have shown that some embryos derived from abnormally fertilized oocytes can lead to the birth of normal offspring through embryo transfer, in situations where sufficient embryos from normal 2PN sources are available, abnormally fertilized embryos are still discarded. Multiple mechanisms prevent polyspermy to ensure normal fertilization. However, despite these safeguards, the formation of 3PN zygotes can still occur in some cycles undergoing conventional *in vitro* fertilization (C-IVF). According to reports, the incidence of 3PN zygotes during C-IVF ranges from 2% to 10% (7).

The oocyte is widely recognized as the primary determinant of embryonic developmental potential (8). Maternal age is a pivotal factor contributing to the decline in fertility and adverse reproductive outcomes (9–11). Women over the age of 35 years experience reduced fertility, and their oocytes are more prone to errors in meiotic chromosome segregation, resulting in oocytes with an incorrect number of chromosomes, a condition known as aneuploidy (9–11). When using IVF, there is no significant difference in the live birth rate per oocyte retrieval cycle and per embryo transfer cycle among male partners of various age groups when the female partner is younger than 35 years old or falls within the 40–44 age group. However, when the female partner is aged between 35 and 39, a trend of decreasing live birth rate is observed as the male partner's age increases (12).

Both sperm and oocyte factors are pivotal in ensuring successful fertilization and subsequent embryonic development. Individuals who exhibit a favorable response to ovarian stimulation are more prone to experiencing 3PN zygotes. It has been established that the occurrence of 3PN zygotes is linked to a heightened response to gonadotropin stimulation, often evidenced by an elevated number of retrieved oocytes in patients undergoing ICSI (13). One of our previous studies, which focused on female patients younger than 38 years old undergoing C-IVF with donor sperm (C-IVFD), revealed that more retrieved oocytes and higher peak estradiol (E_2_) level on the day of human chorionic gonadotrophin (hCG) administration are risk factors for the incidence of 3PN zygotes (14). Another study, which included female patients younger than 40 years old undergoing C-IVF with partner's sperm, demonstrated that there was no significant difference in the number of retrieved oocytes between the 3PN = 0% group and the 3PN > 0% group (15).

To minimize the potential confounding effects of factors from maternal age and partner's semen quality, this study included women under 35 years old and cycles involving donor sperm for *in vitro* fertilization, and aimed to investigate the effect of 3PN incidence on pregnancy outcomes in women with a favorable prognosis undergoing conventional *in vitro* fertilization with donor sperm-embryo transfers (C-IVFD-ETs).

## 2 Materials and methods

### 2.1 Study design

A retrospective cohort study was conducted in accordance with the Strengthening the Reporting of Observational Studies in Epidemiology (STROBE) guidelines for observational studies. This study was approved by the Ethics Review Board of Northwest Women's and Children's Hospital (approval number: 2023003).

### 2.2 Setting, participants and variables

This study included first-time C-IVFD-ETs cycles with fresh embryo transfer performed at Northwest Women's and Children's Hospital from January 2014 to January 2021.

The inclusion criteria were as follows: (1) women with age ≤ 35 years; (2) receiving long/ultra-long agonist protocol ovarian stimulation; and (3) first-time C-IVFD-ETs cycles. The exclusion criteria were: (1) history of oocyte *in vitro* maturation; (2) previous uterine or ovary surgery; (3) presence of uterine malformation; and (4) undergoing ovarian stimulation with antagonist, short or ultra-short protocol.

**Figure 1:**
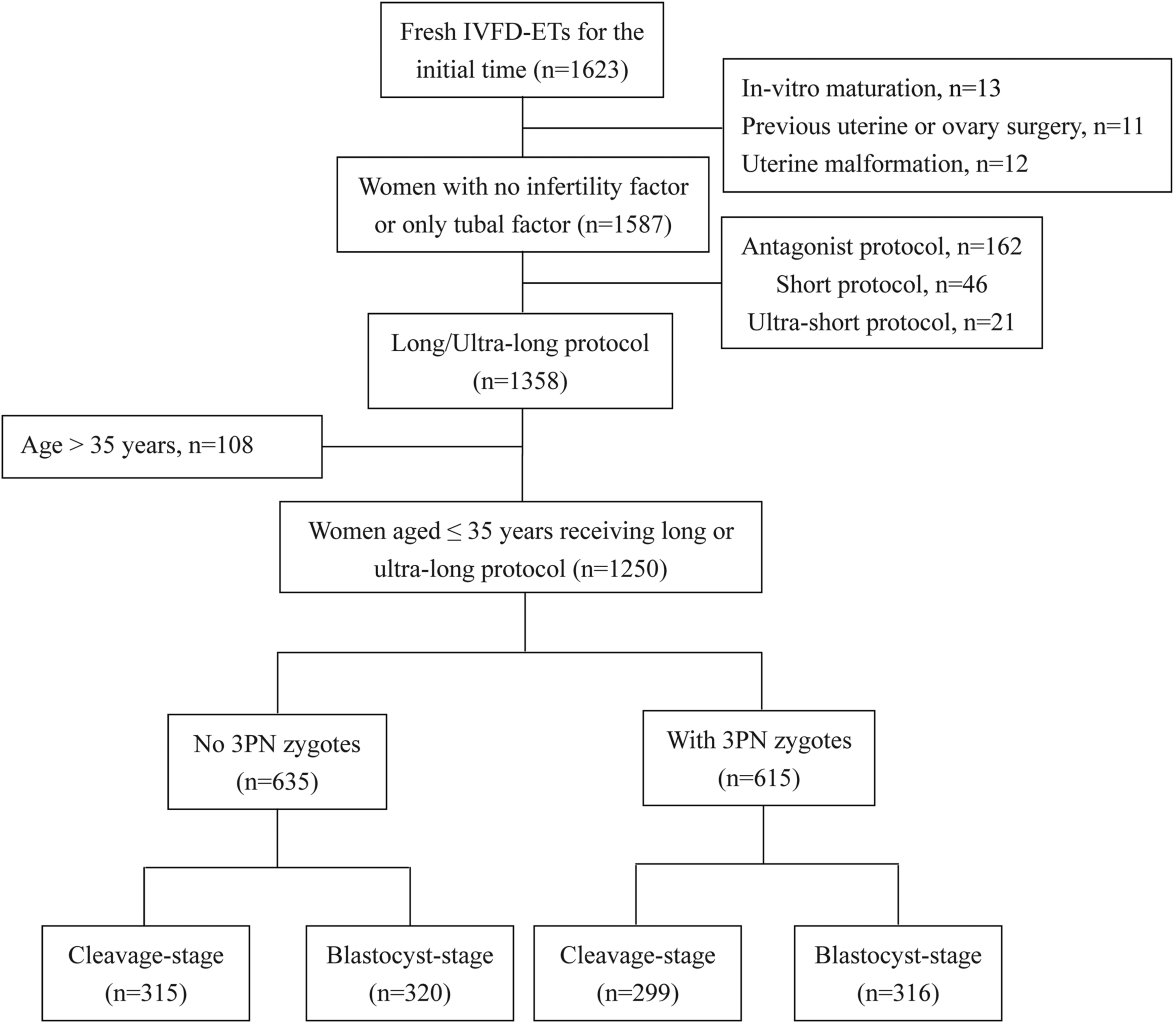
Study flow chart.

As illustrated in Figure 1, a total of 1,250 cycles were included and categorized into two groups: the 3PN = 0% group, consisting of cycles with no 3PN zygotes (*n* = 635), and the 3PN > 0% group, comprising cycles with the presence of 3PN zygotes (*n* = 615).

### 2.3 Outcomes

The primary outcomes were clinical pregnancy, ongoing pregnancy, abortion and live birth rates. The secondary outcomes were cleavage, high-quality embryo, available embryo, implantation, and ectopic pregnancy rates.

### 2.4 Ovarian stimulation protocol

All ovarian stimulation cycles were conducted using first-attempt down-regulated protocols (long or ultra-long protocols) with gonadotropin-releasing hormone agonist (GnRH-a; Decapeptyl®, Ferring Pharmaceuticals, Germany), followed by controlled ovarian hyperstimulation using follicle-stimulating hormone (FSH) preparations. The stimulation protocol employed either recombinant FSH (Gonal-F®, Merck Serono, Switzerland), urinary-derived FSH (Puregon®, N.V. Organon, Netherlands; Urofollitropin®, Livzon Pharmaceutical, China), or human menopausal gonadotropin (hMG®, Livzon Pharmaceutical, China) containing both FSH and luteinizing hormone activity. The daily dosage of stimulation medication was decided and adjusted based on patient characteristics and treatment response to achieve optimal ovarian stimulation. When more than 3 follicles exceeded 18 mm in size, a dose of 10,000 units of hCG was administered. Subsequently, oocyte retrieval was performed 36 h later by a transvaginal ultrasonography-guided aspiration process.

### 2.5 Origin of sperm and C-IVFD procedure

After retrieval, the oocyte-cumulus complexes (OCCs) were cultured in a specialized medium (G-IVF; Vitrolife, Sweden). Conventional *in vitro* fertilization with donor sperm (C-IVFD) was performed using semen from donor. Semen samples were obtained from Shaanxi Province Human Sperm Bank. The general procedures for donor recruitment, sperm freezing, and recipient selection followed the standards of National Health and Family Planning Commission (NHFPC) of the People's Republic of China. The semen parameters for donors adhered to the National Sperm Bank (before freezing: volume ≥2.0 ml, concentration ≥60 million/ml, progressive motility ≥60%, normal sperm morphology ≥70%; after thawing: concentration ≥15 million/ml, progressive motility ≥32%, normal sperm morphology ≥4%) (16). Semen samples were processed using a discontinuous density gradient centrifugation protocol (40% and 80% gradients; Sage, Costa Rica). Primary centrifugation was performed at 200 ×  g for 20 min at room temperature. Following supernatant removal, the resulting pellet was resuspended in sperm washing medium (Spermrinse; Vitrolife, Sweden) and subjected to secondary centrifugation at 200 ×  g for 5 min. Final sperm preparation was adjusted to achieve a concentration of 100,000 motile spermatozoa per 0.6 ml fertilization medium for oocyte co-incubation.

### 2.6 Embryo culture and assessment

All culture media were overlaid with paraffin oil (Ovoil; Vitrolife, Sweden) and equilibrated in a humidified atmosphere at 37°C for at least 24 h before use. 5 h after C-IVFD insemination, the zygotes were transferred to a cleavage medium (G-1 plus; Vitrolife, Sweden). Sixteen to twenty hours post-fertilization, the fertilization status was observed under an inverted microscope by confirming the presence of two polar bodies in the perivitelline space and the pronuclei within the zygote. The number of pronuclei (PN) in each zygote was recorded: zygotes with two pronuclei (2PN) were considered normally fertilized; those with a single pronucleus (1PN) or ≥3PN were classified as abnormally fertilized. The occurrence of zygotes with 3PN was specifically documented. On day 3, only the embryos derived from 2PN zygotes designated for blastocyst culture were subsequently transferred to a blastocyst medium (G-2 plus; Vitrolife, Sweden), where they remained until day 6. The cleavage-stage embryos were evaluated based on the homogeneity of blastomeres, the number of blastomeres, and the degree of embryo fragmentation. High-quality cleavage-stage embryos were classified as graded I or II. The available cleavage-stage embryos were graded I, II, and III (17). The blastocyst-stage embryos were graded according to the method proposed by Gardner, with scores assigned based on their developmental stage ranging from 1 to 6, the quality of the inner cell mass, and the trophectoderm (18). High-quality blastocysts were defined as those graded ≥3BB.

Most of the embryos were cultured in conventional incubators. A small fraction of embryos, primarily from patients with a higher number of retrieved oocytes or previous unsuccessful cycles, were cultured in an EmbryoScope time-lapse device (Vitrolife, Sweden). The 16-well plates were prepared the day before insemination. Two 180 μl drops of cleavage medium were placed in an EmbryoSlide+(Vitrolife, Sweden) covered with 1.4 ml of mineral oil (Vitrolife, Sweden). These plates were maintained at 37°C, under 6% CO_2_, 5% O_2_, and 89% N_2_ atmosphere. Images of each embryo were captured every 20 min in seven different focal planes throughout the culture period and were later analyzed using the EmbryoViewer external image analysis software (Vitrolife, Sweden). Embryos with multinucleation at the 2-cell stage (MN2) were specifically documented through time-lapse monitoring.

### 2.7 Embryo transfer and pregnancy confirmation

In our center, a maximum of two embryos were transferred in each cycle. The cleavage-stage embryo was transferred on day 3, while the blastocyst-stage embryo was transferred on day 5 or 6. Patients were given 60 mg progesterone (Xianju Pharmaceutical Co.,Ltd.; Zhejiang, China) via intramuscular injection or 90 mg of vaginal progesterone gel (Crinone®, Merck Serono, Switzerland) daily following embryo transfer for 10 weeks. An ET catheter (Cook Ireland Ltd., Limerick, Ireland) was utilized to place the embryos with the guidance of transabdominal ultrasound. To increase the success rate of embryo transfer, the mucus at the cervical os was removed beforehand with a cotton swab soaked in warm saline solution (19). Serum *β*-hCG levels were measured 14 days after cleavage-stage embryo transfer and 12 days after blastocyst transfer. Clinical pregnancy was defined as the presence of an intrauterine gestational sac on ultrasonography during the first trimester. Ongoing pregnancy was defined as a clinical pregnancy that continued for at least 12 weeks. Abortion was defined as loss of clinical pregnancy before 24 weeks. Live birth was defined as a pregnancy resulting in the birth of at least a live infant.

### 2.8 Statistical analysis

Statistical analysis between groups for continuous variables was performed with *Student's t*-test for data with normal distribution. A non-parametric *Mann–Whitney U-test* was performed for data with skewed distribution. Statistical analysis between groups for categorical variables was expressed as number and percentage and *Chi-square* test or *Fisher exact* test was performed. Forward logistic regression analysis was performed to determine the risk factors for 3PN incidence. The statistical analysis was performed with SPSS version 23 (IBM Corp.; NY, USA). *p* < 0.05 was considered statistically significant.

## 3 Results

### 3.1 The characteristics of participants

The flow chart of the study is shown in Figure 1. Ultimately, a total of 1,250 cycles were included. The 1,250 cycles were divided into two groups: the 3PN = 0% group (*n* = 635, 50.8%) and the 3PN > 0% group (*n* = 615, 49.2%). The general and laboratory data of patients are shown in Table 1.

**Table 1:** The general characteristics and laboratory data.

Variables	3PN = 0%	3PN > 0%	*p-*value
No. transfers	635	615	/
Age, years	28.27 ± 3.09	27.93 ± 3.13	0.053
BMI, kg/m^2^	22.15 ± 3.04	22.02 ± 2.98	0.217
Basal FSH (mIU/ml)	6.87 ± 2.55	6.67 ± 1.83	0.110
Basal E_2_ (pg/ml)	46.13 ± 24.67	46.05 ± 22.29	0.947
Total Gn dosage (IU)	2,081.80 ± 782.32	2,024.37 ± 707.53	0.185
Gn stimulation, days	10.50 ± 2.36	10.79 ± 7.26	0.348
Peak E_2_ level on hCG administration day (pg/ml)	4,240.57 ± 2,125.18	4,638.49 ± 2,242.27	<0.001*
No. of retrieved oocytes (*n*)	10.19 ± 4.24	11.87 ± 4.20	<0.001*
Infertility type (%, *n*)			0.699
Primary infertility	77.8 (494)	78.7 (484)	
Secondary infertility	22.2 (141)	21.3 (131)	
Endometrial thickness (mm)	12.05 ± 2.57	12.08 ± 2.53	0.626
No. transferred embryos = 1	291	293	0.425
Cleavage-stage (%, *n*)	18.9 (55)	16.4 (48)	
Blastocyst-stage (%, *n*)	81.1 (236)	83.6 (245)	
No. transferred embryos =2	344	322	0.470
Cleavage-stage (%, *n*)	75.6 (260)	78.0 (251)	
Blastocyst-stage (%, *n*)	24.4 (84)	22.0 (71)	
Normal fertilization (%, *n*)	69.3 (4,481/6,470)	62.9 (4,576/7,279)	<0.001*
Cleavage (%, *n*)	97.1 (4,351/4,481)	96.6 (4,422/4,576)	0.205
High-quality embryo (%, *n*)	56.7 (2,465/4,351)	56.6 (2,504/4,422)	0.979
Available embryo (%, *n*)	86.9 (3,782/4,351)	88.2 (3,902/4,422)	0.061

BMI, body mass index; FSH, follicle-stimulating hormone; E_2_, estradiol; Gn, gonadotropin; hCG, human chorionic gonadotropin.

* *p* < 0.05 was regarded as statistically significant.

### 3.2 The influencing factors of 3pn incidence

The multivariate logistic regression analysis was performed to determine the incidence of 3PN zygotes, considering potential factors such as age, body mass index (BMI), basal FSH, basal E_2_, peak E_2_ level on hCG administration day, total gonadotropin (Gn) dosage, Gn stimulation time, and the number of retrieved oocytes serving as independent variables. It was observed that the peak E_2_ level on the day of hCG administration (OR: 1.16, 95% CI 1.12–1.19, *p* < 0.001) and the number of retrieved oocytes (OR: 1.08, 95% CI 1.05–1.11, *p* < 0.001) were independently associated with 3PN incidence (Table 2).

**Table 2:** Multivariate logistic regression analysis of risk factors for 3PN incidence.

Variables	OR	95% CI	*p-*value
Age, years	0.97	0.94–1.01	0.143
BMI, kg/m^2^	0.98	0.94–1.02	0.302
Basal FSH (mIU/ml)	0.98	0.93–1.04	0.477
Basal E_2_ (pg/ml)	1.00	0.99–1.00	0.782
Peak E_2_ level on hCG administration day (pg/ml)	1.16	1.12–1.19	<0.001*
Total Gn dosage (IU)	1.00	1.00–1.00	0.950
Gn stimulation, days	1.01	0.98–1.04	0.561
No. of retrieved oocytes (*n*)	1.08	1.05–1.11	<0.001*

CI, confidence interval; OR, odds ratio; BMI, body mass index; FSH, follicle-stimulating hormone; E_2_, estradiol; Gn, gonadotropin; hCG, human chorionic gonadotropin.

* *p* < 0.05 was regarded as statistically significant.

### 3.3 The impact of 3pn on pregnancy outcomes

Regardless of the transfer stage, no significant differences were observed in the rates of cleavage (97.1% vs. 96.6%, *p* *=* 0.205), high-quality embryos (56.7% vs. 56.6%, *p* *=* 0.979), available embryos (86.9% vs. 88.2%, *p* *=* 0.061), implantation (59.4% vs. 56.6%, *p* = 0.201), clinical pregnancy (72.9% vs. 68.5%, *p* = 0.083), or ectopic pregnancy (0.6% vs. 0.5%, *p* = 0.732) between the 3PN = 0% group and 3PN > 0% group, as detailed in Tables 1, 3. A significant increase was observed in ongoing pregnancy rates (68.5% vs. 62.8%, *p* = 0.033) and live birth rates (66.9% vs. 59.8%, *p* = 0.009) in the 3PN = 0% group compared to those in the 3PN > 0% group. The abortion rate in the 3PN = 0% group was significantly lower than that in the 3PN > 0% group (7.8% vs. 12.3%, *p* = 0.026) (Table 3).

**Table 3:** Comparison of pregnancy outcomes between the 3PN = 0% and 3PN > 0% groups.

Parameter	Cleavage-stage		*p-*value	Blastocyst-stage		*p-*value	Total		*p-*value
	3PN = 0%	3PN > 0%		3PN = 0%	3PN > 0%		3PN = 0%	3PN > 0%	
No. transfers	315	299	/	320	316	/	635	615	/
Implantation (%, *n*)	52.5 (302/575)	50.5 (278/550)	0.507	69.3 (280/404)	65.1 (252/387)	0.229	59.4 (582/979)	56.6 (530/937)	0.201
Clinical pregnancy (%, *n*)	71.4 (225/315)	67.2 (201/299)	0.259	74.4 (238/320)	69.6 (220/316)	0.182	72.9 (463/635)	68.5 (421/615)	0.083
Ongoing pregnancy (%, *n*)	67.9 (214/315)	62.2 (186/299)	0.137	69.1 (221/320)	63.3 (200/316)	0.124	68.5 (435/635)	62.8 (386/615)	0.033*
Twin pregnancies (%, *n*)	34.2 (77/225)	37.8 (76/201)	0.441	17.6 (42/238)	14.5 (32/220)	0.368	25.7 (119/463)	25.7 (108/421)	0.871
Ectopic pregnancy (%, *n*)	1.3 (3/225)	0.5 (1/201)	0.372	0.0 (0/238)	0.5 (1/220)	0.298	0.6 (3/463)	0.5 (2/421)	0.732
Abortion (%, *n*)	7.6 (17/225)	11.4 (23/201)	0.17	8.0 (19/238)	13.1 (29/220)	0.070	7.8 (36/463)	12.3 (52/424)	0.026*
Live birth (%, *n*)	66.0 (208/315)	59.5 (178/299)	0.096	67.8 (217/320)	60.1 (190/316)	0.044**	66.9 (425/635)	59.8 (368/615)	0.009*

* *p* < 0.05 was regarded as statistically significant.

When the results were analyzed based on embryo transfer stage, we observed that among patients who underwent cleavage-stage embryo transfer, there were no significant differences in pregnancy outcomes between the two groups (Table 3). However, in patients undergoing blastocyst-stage embryo transfer, the live birth rate was significantly higher in the 3PN = 0% group compared to the 3PN > 0% group (67.8% vs. 60.1%, *p* = 0.044) (Table 3).

**Figure 2:**
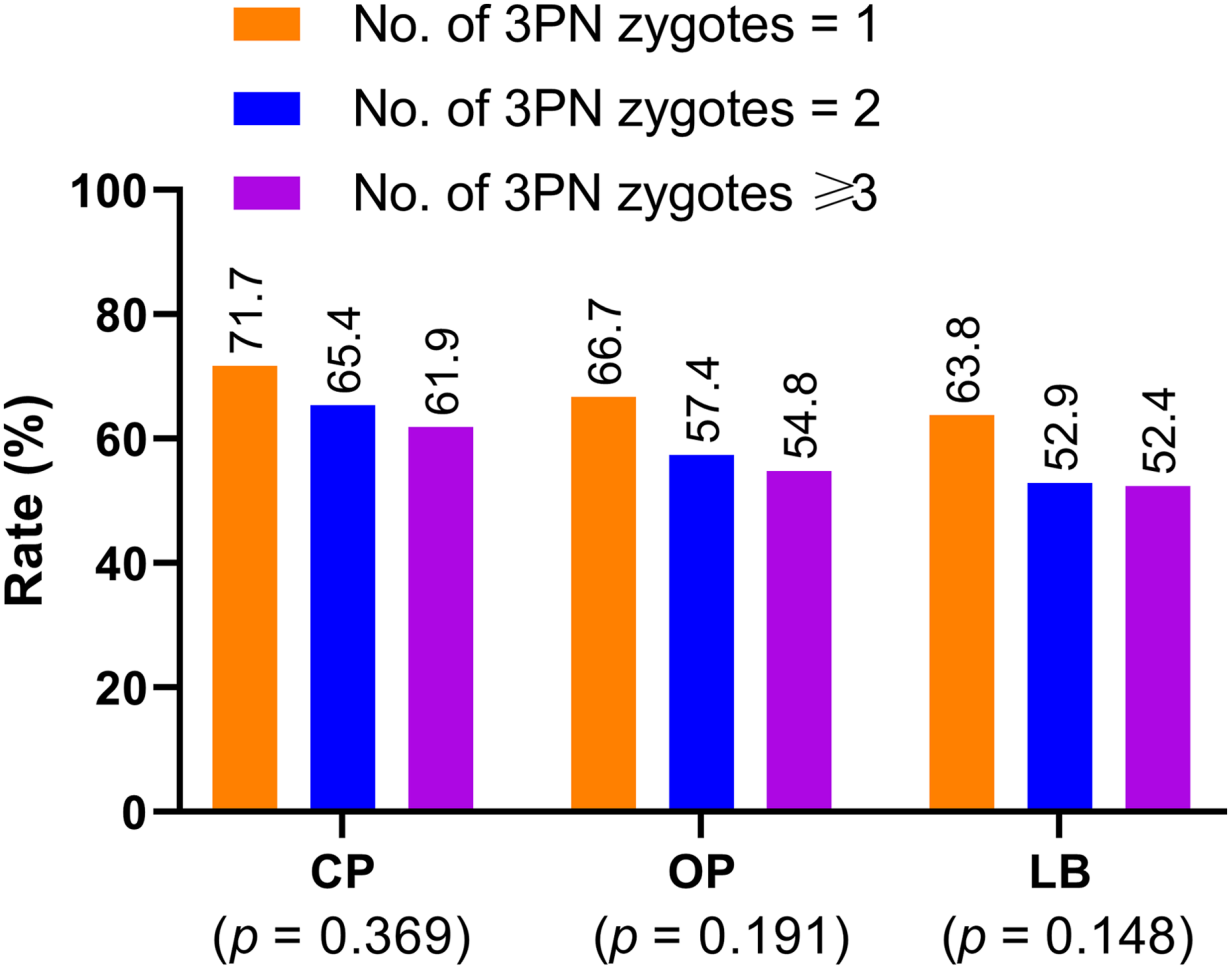
Clinical pregnancy, ongoing pregnancy, and live birth rates in patients undergoing blastocyst-stage embryo transfer with embryos derived from zygotes with different 3PN numbers. CP: clinical pregnancy; OP: ongoing pregnancy; LB: live birth.

Additionally, our data demonstrated that the clinical pregnancy (*p* = 0.369), ongoing pregnancy (*p* = 0.191), and live birth rates (*p* = 0.148) decreased as the number of 3PN zygotes increased for patients undergoing blastocyst-stage embryo transfer, but no significant differences were observed (Figure 2).

By using time-lapse monitoring, 180 normal embryos from the 3PN = 0% group and 157 normal embryos from the 3PN > 0% group were analyzed and documented. Although the MN2 rate was slightly higher in the 3PN > 0% group than in the 3PN = 0% group, the difference was not statistically significant (23.6% vs. 16.1%, *p* = 0.085).

## 4 Discussion

Multiple mechanisms prevent polyspermy and ensure normal fertilization (20–22). However, polyspermy safeguards are imperfect, as evidenced by the persistent occurrence of 3PN zygotes in C-IVFD cycles. The causes of 3PN are diverse and primarily attributed to oocyte-derived meiotic failure and polyspermic fertilization (23). In the present study, the use of rigorously screened donor sperm minimized confounding effects of male factors.

Comparing outcomes between donor sperm cycles, partner sperm cycles, and ICSI cycles offers valuable insights. In ICSI, sperm-oocyte interaction is bypassed, theoretically eliminating polyspermy. However, ICSI does not fully prevent 3PN formation, as oocyte activation defects or retention of the second polar body can mimic triploidy (23). Studies report lower 3PN rates in ICSI (2%–5%) compared to C-IVF (2%–10%) (7), yet ICSI cycles with 3PN zygotes still exhibit poorer outcomes, akin to our findings (24, 25). Conversely, partner sperm cycles may introduce confounding variables like sperm DNA fragmentation, which exacerbates fertilization abnormalities (26). Future research should directly compare C-IVF (donor vs. partner sperm) and ICSI cohorts to delineate the roles of sperm competence vs. fertilization technique in 3PN etiology.

Physiological mechanisms linking ovarian response to 3PN risk were further elucidated. Patients exhibiting robust pharmacological responses often yield higher proportions of immature or post-mature oocytes, elevating 3PN susceptibility. Immature oocytes may inadequately execute cortical and zona reactions, while post-mature oocytes exhibit impaired cortical granule release, both increasing polyspermy risk. One of our previous studies, which focused on female patients under 38 years of age undergoing C-IVFD, revealed that more retrieved oocytes and higher peak E_2_ level on hCG administration day are risk factors for the incidence of 3PN zygotes (14). Our data in the present study further corroborated this observation (14).

Other potential contributors warrant exploration. Firstly, genetic predispositions, such as mutations in cortical granule-related genes (e.g., *JUNO* or *ovastacin*), may impair the cortical reaction, increasing susceptibility to polyspermy (20, 27). Secondly, laboratory conditions, including suboptimal sperm-oocyte incubation ratios or variations in culture media composition, could inadvertently promote abnormal fertilization (28, 29). Thirdly, environmental factors, such as oxidative stress during oocyte maturation, may compromise zona pellucida integrity, further predisposing to polyspermy (30). Future studies should investigate these variables to refine protocols and mitigate 3PN incidence.

Several studies analyzed the impact of 3PN on pregnancy outcomes in women undergoing ICSI. Figueira et al. (24) found that cycles with a 3PN incidence greater than 25% had a 3.5-fold higher risk of miscarriage. Dayal et al. (25) also reported a statistically significant inverse correlation between a high proportion of 3PN zygotes and clinical pregnancy rates. Rosen et al. (31) further demonstrated that 3PN formation was a significant negative predictor of implantation. One of our previous studies, which included 509 early rescue- ICSI cycles showed that there was no significant difference in good quality embryo, available embryo, implantation, clinical pregnancy and live birth rates between the 3PN = 0% and 3PN > 0% groups. However, the 3PN = 0% group showed significantly lower abortion than that in the 3PN > 0% group (32). Our present study reported the impact of 3PN on pregnancy outcomes in women undergoing C-IVFD, and demonstrated that the absence of 3PN zygotes was associated with significantly higher ongoing pregnancy rates and live birth rates, alongside a lower abortion rate.

The observed reduction in ongoing pregnancy and live birth rates in cycles with 3PN zygotes may stem from downstream embryological consequences. Even when embryos derived from 2PN zygotes are transferred, the presence of 3PN zygotes in the cohort may reflect broader oocyte quality issues. Aneuploidy, mitochondrial dysfunction, or epigenetic anomalies in sibling oocytes could impair embryonic developmental competence (9, 33). Additionally, time-lapse monitoring revealed a trend toward higher MN2 rates in the 3PN > 0% group, suggesting that mitotic errors in ostensibly normal embryos may contribute to implantation failure or early pregnancy loss (34, 35). These findings align with studies linking 3PN cycles to increased embryonic mosaicism and reduced blastocyst euploidy (27, 36). Thus, 3PN incidence may serve as a biomarker for suboptimal oocyte quality, indirectly affecting pregnancy outcomes even in transferred embryos.

MN2, a cytokinesis-related nuclear abnormality, may further elucidate 3PN-associated outcomes (37). While prior studies proposed a link between high 3PN proportions and MN2 incidence (38), our time-lapse data showed no statistically significant difference in MN2 rates between groups, though limited by sample size. Cycles with robust ovarian responses (elevated E_2_, high oocyte yield) are prone to MN2 (39, 40), a phenomenon potentially masked in conventional incubators due to restricted observational windows (34, 41). Notably, cleavage-stage transfers in our cohort showed comparable outcomes regardless of 3PN status, possibly because MN2 typically occurring approximately 43 h post-fertilization, escapes detection in day-3 assessments.

Egashira et al. (42) demonstrated that blastocysts derived from MN2 embryos exhibit similar implantation potential to those derived from normal embryos. Balakier et al. (43) proposed that the majority of MN2 embryos have the capacity to self-correct during early cleavage divisions and can develop into euploid blastocysts, ultimately leading to the birth of healthy infants. These findings suggest that extending the culture of MN2 embryos to the blastocyst stage before transfer could potentially improve pregnancy outcomes. However, our data revealed significantly lower live birth rates in blastocyst transfers from the 3PN > 0% group, suggesting that 3PN presence signals intrinsic embryo cohort deficiencies unmitigated by extended culture. This underscores the need for comprehensive embryo selection strategies integrating genetic and morphokinetic assessments.

### 4.1 The strengths and limitations of the study

One of the primary strengths of this study lies in its large sample size, which enhances the reliability of the findings. Furthermore, the use of donor sperm allowed us to minimize the impact of abnormal sperm on reproductive outcomes, thereby isolating the effects of oocyte quality. Recognizing the pivotal role of oocyte quality in embryo development, we specifically targeted IVD-ETs for our analysis. Moreover, conducting the study at a single center ensured that the embryos were cultured in uniform media and graded according to consistent criteria, thereby eliminating potential biases introduced by variations in culture media and techniques.

This study has certain limitations that should be acknowledged. Firstly, its retrospective design represents a major constraint. By focusing solely on fresh cycles, we were unable to factor in the detrimental effects of controlled ovarian hyperstimulation on endometrial receptivity. Secondly, despite observing a potential link between the frequency of MN2 events and the occurrence of 3PN zygotes, the relatively limited sample size used for time-lapse monitoring may have hindered our capacity to detect statistically significant differences in reproductive outcomes.

## 5 Conclusions

In conclusion, the peak E_2_ level on hCG administration day and the number of retrieved oocytes were independently associated with the presence of 3PN zygotes in a cohort of fertilized oocytes. The incidence of 3PN zygotes had a negative influence on pregnancy outcomes in women with a good prognosis undergoing C-IVFD-ETs.

## Data Availability

The raw data supporting the conclusions of this article will be made available by the authors, without undue reservation.
